# Effective Removal of Sulfonamides Using Recyclable MXene-Decorated Bismuth Ferrite Nanocomposites Prepared via Hydrothermal Method

**DOI:** 10.3390/molecules28041541

**Published:** 2023-02-05

**Authors:** Pascaline Sanga, Juanjuan Wang, Xin Li, Jia Chen, Hongdeng Qiu

**Affiliations:** 1 CAS Key Laboratory of Chemistry of Northwestern Plant Resources and Key Laboratory for Natural Medicine of Gansu Province, Lanzhou Institute of Chemical Physics, Chinese Academy of Sciences, Lanzhou 730000, China; 2 University of Chinese Academy of Sciences, Beijing 100049, China; 3 College of Chemistry, Zhengzhou University, Zhengzhou 450001, China

**Keywords:** MXene, bismuth ferrite, nanocomposite, sulfonamide, adsorption

## Abstract

Developing a simple and efficient method for removing organic micropollutants from aqueous systems is crucial. The present study describes the preparation and application, for the first time, of novel MXene-decorated bismuth ferrite nanocomposites (BiFeO_3_/MXene) for the removal of six sulfonamides including sulfadiazine (SDZ), sulfathiazole (STZ), sulfamerazine (SMZ), sulfamethazine (SMTZ), sulfamethoxazole (SMXZ) and sulfisoxazole (SXZ). The properties of BiFeO_3_/MXene are enhanced by the presence of BiFeO_3_ nanoparticles, which provide a large surface area to facilitate the removal of sulfonamides. More importantly, BiFeO_3_/MXene composites demonstrated remarkable sulfonamide adsorption capabilities compared to pristine MXene, which is due to the synergistic effect between BiFeO_3_ and MXene. The kinetics and isotherm models of sulfonamide adsorption on BiFeO_3_/MXene are consistent with a pseudo-second-order kinetics and Langmuir model. BiFeO_3_/MXene had appreciable reusability after five adsorption–desorption cycles. Furthermore, BiFeO_3_/MXene is stable and retains its original properties upon desorption. The present work provides an effective method for eliminating sulfonamides from water by exploiting the excellent texture properties of BiFeO_3_/MXene.

## 1. Introduction

Since the economy and industry have continued to grow, the environment has been severely destroyed, and water pollution has become a serious problem. Protecting water resources and preventing water pollution have become increasingly important in modern society. For example, the presence of organic pollutants in the aquatic environment, such as personal-care products, pharmaceuticals, steroid hormones, industrial chemicals, and pesticides, has caused great concern. It is known that sewage treatment plants, hospitals, and medical wastewater produce organic pollutants in aquatic environments when they are not properly treated [1,2,3,4]. Due to their toxicity to water ecosystems and human health, the presence of these pollutants in wastewater is considered an environmental threat [5]. Sulfonamides are synthetic medicines used to treat and prevent a variety of human and animal diseases [6,7]. Moreover, the use of sulfonamides over a prolonged period or in excess results in their widespread release into the environment, increasing aquatic microorganism resistance, which, in turn, will adversely affect human health through the food chain in future generations [8,9]. In severe cases, it may lead to an imbalance in the body’s flora, a malfunction of the digestive system, and even death [10,11]. In addition to posing a serious threat to human health, the ecosystem, as well, is seriously threatened. Therefore, a simple, efficient, and stable method must be developed to remove sulfonamide antibiotics from water. To date, several techniques have been used to remove antibiotics from the environment, including biodegradation, oxidation, adsorption, and photocatalysis, etc. [12,13,14,15] In comparison to other methods, adsorption is a simple and effective method with low costs, high levels of efficiency, low waste, and environmental-protection characteristics [16,17]. Choosing the right adsorbent is of critical importance, since it directly affects the adsorption capacity, removal efficiency, and selectivity of the process.

MXenes are two-dimensional transition-metal carbides, carbonitrides, and nitrides that can be synthesized by etching the intermediate A layers of an M_n+1_AX_n_ phase, where M stands for early transition metal (such as Ti, Cr, V, Nb, etc.), A is an element of group A (such as Al, Si, Sn, etc.), X is carbon or nitrogen, and n equals 1, 2 or 3 [18,19,20,21,22]. The remarkable properties of MXenes, such as their extraordinary morphologies, superhydrophobic surface, high metallic conductivity, and exceptional mechanical properties, make them the most attractive materials for a broad range of applications [23,24,25,26]. It is particularly noteworthy that functional groups with tunable end groups endow MXenes with controllable properties, which facilitate the development of hybrids with enhanced flexibility [27]. MXenes and similar composites are proven to be outstanding alternative substances with tremendous promise for environmental remediation based on their exceptional performance. Due to their unique morphology, MXene-based materials excel as prospects for environmental applications due to their high hydrophilicity, large specific surface area, controllable layer thickness, adaptable structural design, and diverse composition. Taking advantage of these properties, applications of MXene-based materials in environmental remediation, such as the adsorption and catalytic degradation of pollutants such as dyestuffs, organic compounds, heavy metal ions, oxidative metal ions, radioactive contaminants, antibiotics, and even waste gases, have been thoroughly explored in recent years [23]. However, like most double-dimensional materials, MXene nanosheets are susceptible to self-aggregation due to van-der-Waals interactions as well as hydrogen-bond interactions. This results in a reduction in surface area and the poor performance of the material [28]. Introducing another active material is considered a viable solution to address this issue and improve their performance [27,29]. Furthermore, it has been reported that MXene functionalized with other materials can catalyze dye degradation such as methylene blue (MB) [30], methyl orange (MO) [31], and rhodamine B (RhB) [32]. Therefore, MXene combined with other materials is expected to achieve enhanced performance and excellent stability.

Herein, we reported a versatile approach to preparing a BiFeO_3_/MXene composite for the effective removal of sulfonamides. BiFeO_3_ nanoparticles were grown on the sheets of MXene using the single-step hydrothermal method. We assessed the adsorption performance of BiFeO_3_/MXene on sulfonamides by changing several experimental conditions, including reaction time, concentration of adsorbates, and solution pH. BiFeO₃/MXene has shown a good performance and good reusability for the removal of sulfonamides.

## 2. Results and Discussion

### 2.1. Characterizations of Ti_3_C_2_T_x_ MXene and BiFeO_3_/MXene

**Scheme 1:**
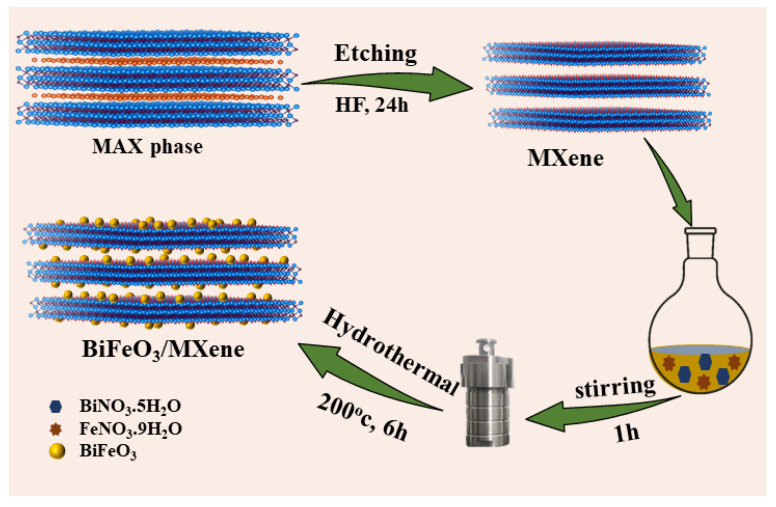
Preparation of BiFeO_3_ nanoparticles on layered MXene.

**Figure 1:**
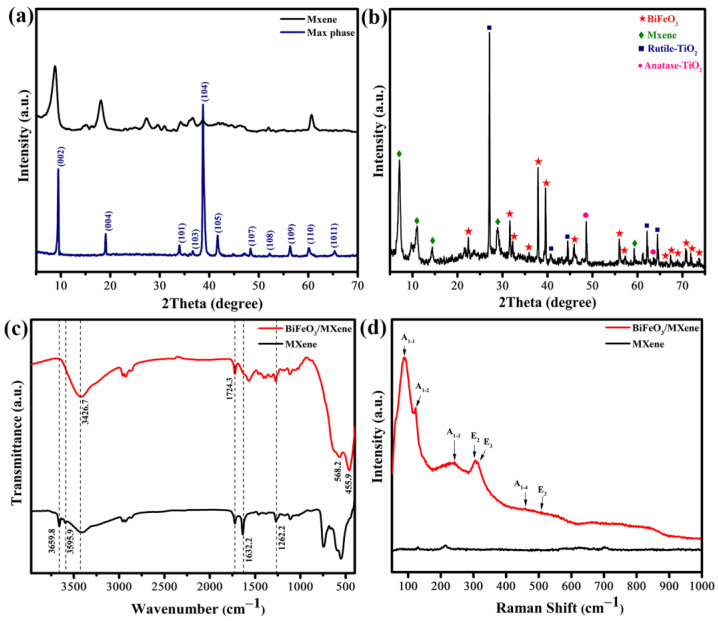
XRD of (**a**) MAX phase and Mxene and (**b**) BiFeO_3_/Mxene, (**c**) FTIR, and (**d**) Raman spectra of Mxene and BiFeO_3_/Mxene.

Ti_3_C_2_T_x_ MXene sheet-like composites were prepared by selectively etching Al from the corresponding MAX phase (Ti_3_AlC_2_) with hydrofluoric acid (HF), as illustrated in Scheme 1. The shift in the (002) peak to a lower angle and the disappearance of the highest diffraction peak of Ti_3_AlC_2_ at 39° (2Ө) in the X-ray diffraction (XRD) patterns suggest that the Ti_3_AlC_2_ was successfully transformed into Ti_3_C_2_T_X_ (Figure 1a). On the obtained Ti_3_C_2_T_X_ was, further, grown BiFeO_3_, using a simple and one-step hydrothermal method. The XRD characterization of the prepared BiFeO_3_/MXene in Figure 1b indicates that the prepared hybrids displayed peaks from MXene and that of BiFeO_3_. In the BiFeO_3_/MXene composite, the characteristic XRD peaks for the BiFeO_3_ phase (PDF number: JCPDS086-1518) can be observed, whereas other peaks of rutile-TiO_2_ and anatase-TiO_2_ were detected as a result of MXene heating above 180 °C [33]. In addition, the appearance of the peak at 10.7° in both BiFeO_3_/MXene and MXene should be ascribed to the corresponding MAX stack structure remaining after the etching process [34].

Fourier transmission infrared (FTIR) spectroscopy was used to determine the chemical structure and functional groups of the prepared materials, as shown in Figure 1c. All spectra showed the peaks at 1724.3 and 1262 cm^−1^ assigned to C=O and C-O stretching vibrations, respectively [34]. The band around 3426.7 cm^−1^ corresponds to the stretching vibration mode of the hydroxyl group (–OH), thus confirming the presence of water molecules. The peaks at 3659.8, 3595.9, and 1632.2 cm^−1^ in Mxene are related to the -OH bending which had been allocated to adsorb H_2_O, confirming to MXene’s hydrophilic character [35]. In addition, the peaks at 568.2 and 455.9 cm^−1^ are indicative of Fe–O stretching and the bending vibrations of octahedral FeO_6_, indicating the existence of the BiFeO_3_ phase [36].

The Raman spectrum of MXene and BiFeO_3_/MXene are presented in the 50–1000 cm^−1^ region (Figure 1d). Raman spectroscopy provides information about structural phase transitions and lattice properties. As is well-known, there are 13 Raman active modes predicted for the space group R3c and rhombohedral distorted structure [37]. The obtained spectrum was characterized by 4A1 and 3E modes. The 4A1 modes are located at 88.4, 122.6, 241.2, and 458.7 cm^−1^ while the E modes are located at 305.8, 315.1, and 508.9 cm^−1^. The lower frequency modes (<400 cm^−1^) are associated with the Bi-O covalent bond, whereas the higher frequency modes (>400 cm^−1^) are related to Fe-O bonds [38]. However, compared to the pure MXene phase, except for the presence of new apparent peaks from BiFeO_3_, the hybrid shows widening peaks, revealing the interaction between the two phases.

**Figure 2:**
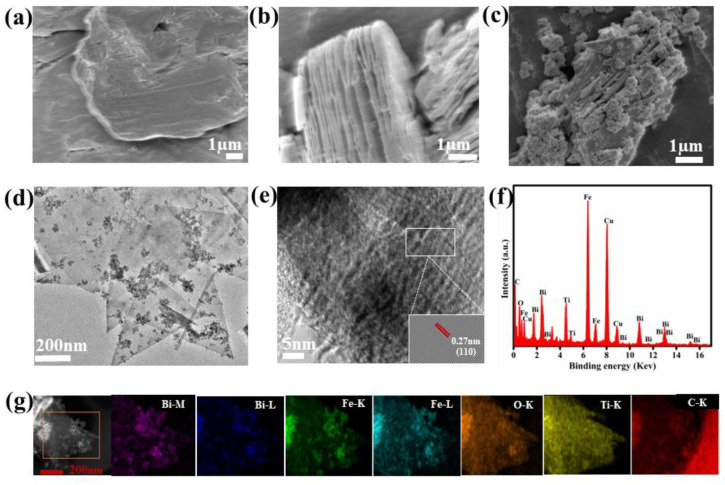
SEM of (**a**) MAX phase, (**b**) MXene, and (**c**) BiFeO_3_/MXene (**d**) TEM, (**e**) HR-TEM, (**f**) EDX spectrum, and (**g**) mapping images of BiFeO_3_/MXene.

Next, the morphological structure and elemental composition of the prepared BiFeO₃/MXene are shown in Figure 2. SEM images in Figure 2a,b, indicate the clear difference between the MAX phase and MXene as a result of Al etching from the MAX phase. In contrast to the BiFeO_3_/MXene composite, pure MXene exhibits the characteristic superposed ultrathin sheet structure (Figure 2b), while the latter is decorated with BiFeO_3_ nanoparticles on both sides of the material’s surface (Figure 2c). Figure 2d presents the typical TEM images of the BiFeO₃/MXene hybrid; it is clear that BiFeO_3_ nanoparticles firmly adhere to the surface of MXene, which is in agreement with the SEM results. The HR-TEM in Figure 2e confirms the crystalline structure of BiFeO_3_, with apparent lattice fringes and a spacing of 0.27 nm corresponding to the (110) plane of the BiFeO_3_ phase [36]. Energy dispersive X-ray (EDX) analysis indicated the presence of Bi, Fe, O, Ti, and C (Figure 2f). It can be seen that the BiFeO_3_ nanoparticles disperse evenly on the MXene nanosheets, as shown in energy-dispersive X-ray spectroscopy (EDX) elemental maps (Figure 2g).

The Brunauer–Emmett–Teller (BET) surface area of MXene was determined to be 3.93 m^2^/g, which increased to 36.24 m^2^/g for BiFeO_3_/MXene. According to the Barret–Joyner–Halenda (BJH) model, the total pore volume of MXene and BiFeO_3_/MXene was 0.021 cm^3^/g and 0.12 cm^3^/g, respectively (Figure S2, see Supplementary Materials). As can be seen, the surface area of BiFeO_3_/MXene significantly increased, by more than ten times compared to pristine MXene. The hybrid sheet structure of BiFeO_3_/MXene presents an open structure that could make it easier for the adsorbate to reach the more active sites in the BiFeO_3_/MXene. Based on these results, it appears that BiFeO_3_ nanoparticles can be grown on MXene using one-step and single-pot techniques, leading to the formation of a BiFeO_3_/MXene hybrid with improved surface area and crystallinity.

**Figure 3:**
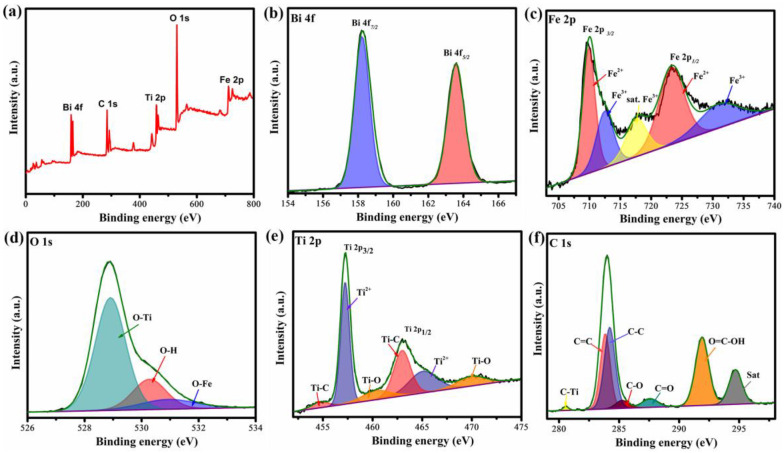
XPS analysis (**a**) survey scan of BiFeO_3_/MXene and high-resolution scans of (**b**) Bi 4f, (**c**) Fe 2p, (**d**) O 1s, (**e**) Ti 2p, and (**f**) C 1s.

The elemental composition and bonding behavior of the BiFeO_3_/MXene were studied using XPS. Figure 3a shows several core peaks of Bi, Fe, O, Ti, and C, indicating that the material is not composed of a single phase. The high-resolution spectrum of Bi 4f (Figure 3b) shows two different characteristic peaks at 158.1 and 163.5 eV, which correspond to the spin-orbit components of Bi 4f_7/2_ and Bi 4f_5/2,_ respectively. According to the literature, this means that bismuth has an oxidation state of 3+ [39,40]. The high-resolution XPS spectrum of Fe 2p (Figure 3c) displayed two main peaks, at 709.8 and 723.4 eV, corresponding to Fe 2p_3/2_ and Fe 2p_1/2_, respectively. These two peaks are known to be associated with the ionic states of Fe. The deconvoluted Fe 2p XPS spectrum shows the appearance of peaks and satellite peaks corresponding to the existence of Fe^3+^ and Fe^2+^ states. The satellite peaks at 717.7 eV are very sensitive to the ionic states of Fe. Therefore, they can also be used to qualitatively determine the oxidation states of Fe. The coexistence of two oxidation states of Fe suggests the presence of oxygen vacancies in the BiFeO_3_/MXene composites, where electron exchange between Fe^3+^-O-Fe^2+^ states may stabilize the charge imbalance in the system that emerged due to oxygen vacancies [41]. The XPS spectra of O 1s (Figure 3d) showed three peaks from the curve deconvolution at 528.8, 530.2, and 531.4 eV, which are ascribed to the lattice oxygen of anatase TiO_2_(O−Ti), hydroxyl oxygen(O−H), and surface-adsorbed oxygen species (O−Fe), respectively [42]. The high-resolution spectrum of Ti 2p represents three doublets (Ti 2p_3/2_ and Ti 2p_1/2_) (Figure 3e). The first, at 454.4 and 463.0 eV, the second, at 457.2 and 465.1 eV, and the third, at 460.3 and 470.2 eV, can be attributed to Ti–C, Ti(II), and Ti–O, respectively [43,44]. The XPS spectra of C 1s (Figure 3f) showed the peaks from the deconvolution located at 280.7, 283.8, 284.2, 285.2, 287.6, 291.8, and 294.7 eV which could be assigned to Ti−C, C=C, C−C, C−O, C=O, O=C−OH, and an interaction satellite, respectively [29,45]. In addition, using XPS elemental composition data (at. %), the ratio of BiFeO_3_: MXene was calculated to be 1:4.

### 2.2. Adsorption Experiments

#### 2.2.1. Adsorption Kinetics

A study of the adsorption kinetics of BiFeO_3_/MXene was conducted to predict their adsorption mechanism toward sulfonamides. The adsorption-kinetic data were investigated using pseudo-first-order and pseudo-second-order models, and intraparticle diffusion models. Pseudo-first-order kinetics describes rate-determining processes such as chemical reactions and mass transport. The pseudo-second-order model assumes that the overall adsorption rate of the adsorbate by the adsorbent is chemisorption, while an intraparticle diffusion model illustrates the adsorption mechanism [46,47,48]. Adsorption kinetics were fitted using the following Equations (1)–(3).

(1) $$\ln(q_{e}-q_{t})=\ln q_{e}-k_{1}t$$

(2) $$\frac{t}{q_{t}}=[\frac{1}{k_{2}q_{e}^{2}}]+\frac{1}{q_{e}}t$$

(3) $$q_{t}=k_{id}t^{\frac{1}{2}}+c$$

where *q_e_* (mg g^−1^) and *q_t_* (mg g^−1^) are the adsorption capacity at equilibrium time and time *t* (min), respectively; *k*_1_ (g mg^−1^ min^−1^) is the pseudo-first-order rate constant; *k*_2_ (g mg^−1^ min^−1^) represents the second-order rate constant; *k_id_* (mg g^−1^⋅min^1/2^) is the intraparticle diffusion rate constant; and *c* (mg g^−1^) is the constant that represents the thickness of the boundary layer.

**Figure 4:**
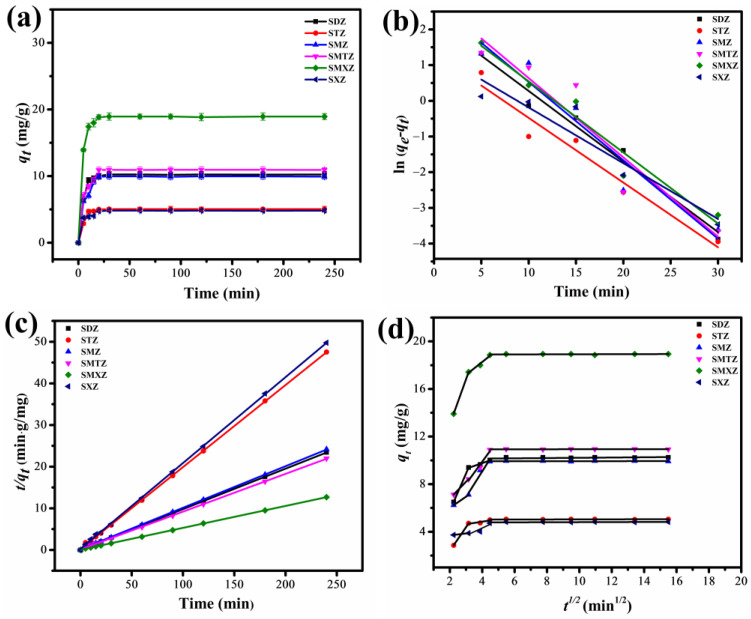
(**a**) Adsorption kinetics of sulfonamides on BiFeO_3_/MXene, (**b**) pseudo-first-order model, (**c**) pseudo-second-order model, and (**d**) intra-particle diffusion model at pH 6.

The results of the adsorption kinetics of sulfonamides on BiFeO_3_/MXene are presented in Figure 4 and the related parameters for the fitted models are listed in Table 1. As shown in Figure 4a, the adsorption for the six sulfonamides was very fast during the first 10 min and then became slow, and, finally, reached the equilibrium in 30 min due to the large specific surface area of BiFeO_3_/MXene. Fast adsorption within 10 min was primarily the result of a greater number of active adsorption sites on the surface BiFeO_3_/MXene, while the slowly adsorbed phase was primarily the result of decreasing adsorption sites and electrostatic repulsion between sulfonamides already adsorbed and those in solution. Nevertheless, to ensure equilibrium, the experiment lasted for 240 min. Figure 4b,c presents the pseudo-first-order and pseudo-second-order models. It can be seen that the pseudo-second-order model fitted much better than the pseudo-first-order model due to their values of *R^2^*. In addition, the theoretical *q_e_* value (11.5, 5.6, 10.9, 13.1, 20.1, and 5.2 mg g^−1^ for SDZ, STZ, SMZ, SMTZ, SMXZ, and SXZ, respectively) was the closest to the experimental data (10.29, 3.82, 10.01, 11.01, 18.99 and 4.84 mg g^−1^ for SDZ, STZ, SMZ, SMTZ, SMXZ, and SXZ, respectively) (Table 1). According to these results, this adsorption is chemisorption rather than mass transport. To investigate the diffusion mechanisms, the experimental data were fitted with the intra-particle diffusion model. The curves in Figure 4d are composed of three linear stages, the presence of multilinearity confirms that intraparticle diffusion is not the only rate-determining step [49]. The first stage involves the rapid diffusion of sulfonamides on the surface of BiFeO_3_/MXene; the second is the diffusion process of sulfonamides molecules into the pore of BiFeO_3_/MXene; the final stage is the chemical bonding which eventually resulted in adsorption equilibrium [50].

#### 2.2.2. Adsorption Isotherms

The adsorption isotherm experiments were conducted to predict the adsorption mechanisms and determine the maximum sulfonamide adsorption capacities of MXene and BiFeO_3_/MXene. To simulate the adsorption isotherms, Langmuir and Freundlich isotherm models were used, and the maximum adsorption capacities were calculated based on the constants obtained from these isotherms. The Langmuir isotherm model represents monolayer adsorption, which implies homogeneous surfaces assuming that all absorption sites possess the same affinity for solutes, whereas the Freundlich isotherm model suggests multilayer adsorption based on the adsorption on heterogeneous sites of adsorbents with varying adsorption energies and affinity [42,47]. Adsorption isotherms were fitted using the Langmuir model and Freundlich model using the following, Equations (4) and (5).

(4) $$\frac{c_{e}}{q_{e}}=\frac{1}{KL}+\frac{c_{e}}{q_{max}}$$

(5) $$\ln q_{e}=KF+\frac{\ln c_{e}}{n}$$

where *q_e_* (mg g^−1^) and *q_max_* (mg g^−1^) represent the equilibrium and maximum adsorption capacity, respectively; *KL* is the Langmuir constant; *KF* is the Freundlich constant; and *n* is the adsorption strength index.

**Figure 5:**
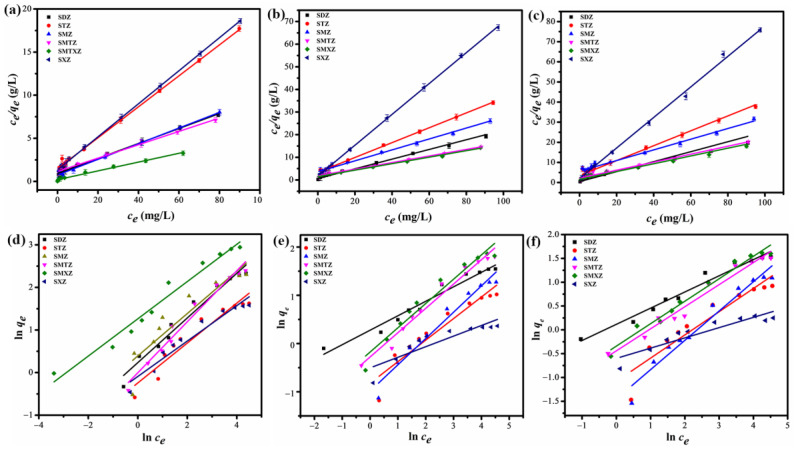
(**a**) Langmuir fitting curves, (**a**) BiFeO_3_/MXene, (**b**) MXene, and (**c**) BiFeO_3_. Freundlich fitting curves (**d**) BiFeO_3_/MXene, (**e**) MXene, and (**f**) BiFeO_3_ for sulfonamides adsorption in a mixed system at pH 6.

The isotherm for the adsorption of sulfonamides on BiFeO_3_/MXene compared to MXene and BiFeO_3_ are shown in Figure 5 and the parameters fitted by the Langmuir and Freundlich models are presented in Table 2. These results show that the Langmuir model fits the experimental data well compared to the Freundlich model due to the relatively high values of the correlation coefficient (*R^2^*). This implies the monolayer adsorption of these sulfonamides onto BiFeO_3_/MXene, and MXene, due to the presence of a surface-terminated functional groups such as -OH, -H, -O and -F. Additionally, according to Freundlich constants (greater than 1), sulfonamides were favorably adsorbed onto BiFeO_3_/MXene, MXene and BiFeO_3_. The calculated values of maximum adsorption capacities (q_max_) in a mixed system show that the BiFeO_3_/MXene composite exhibits a higher adsorption capacity (11.6, 5.6, 10.9, 13.1, 20.1, and 5.2 mg g^−1^ for SDZ, STZ, SMZ, SMTZ, SMXZ, and SXZ, respectively) than MXene (4.9, 3, 4.3, 6.9, 7.1 and 1.5 mg g^−1^ for SDZ, STZ, SMZ, SMTZ, SMXZ, and SXZ, respectively) and BiFeO_3_ (4.9, 2.8, 3.8, 5.2, 5.7, and 1.3 mg g^−1^ for SDZ, STZ, SMZ, SMTZ, SMXZ, and SXZ, respectively) under the same experimental conditions. This may be due to the synergistic effect between BiFeO_3_ and MXene. In addition, the adsorption capacity of BiFeO_3_/MXene on the six sulfonamides was investigated in a single system and the isotherms are shown in Figure S3. The results show that the maximum adsorption capacity was 32.7, 54.4, 41.3, 37.8, 29.9, and 24.8 mg g^−1^ for SDZ, STZ, SMZ, SMTZ, SMXZ, and SXZ, respectively (Table 2). These values are higher than those of mixed-system adsorption due to the competition between different analytes in mixed-system adsorption. Furthermore, Table 3 shows the adsorption capacities reported in the literature using different adsorbent materials. It is seen that BiFeO_3_/MXene had the highest adsorption capacity as a result of its higher surface area as well as higher mesoporous volumes, which may facilitate the adsorption of sulfonamides.

#### 2.2.3. Effect of Initial pH on the Adsorption Performance

**Figure 6:**
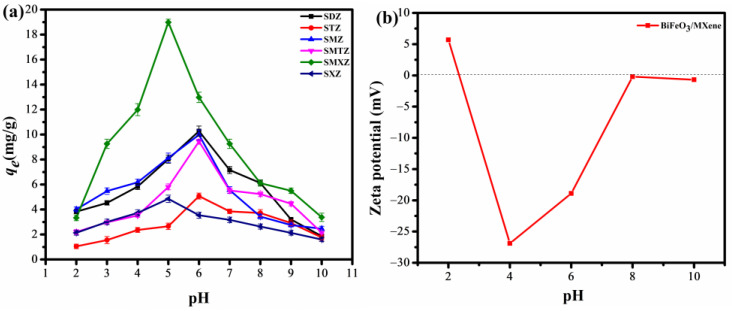
(**a**) Effect of pH on adsorption capacity of sulfonamides, (**b**) zeta potential of BiFeO_3_/MXene.

The pH of the solution can have a significant impact on the chemical characteristics and structure of the adsorbent, as well as on the adsorbate itself, during the adsorption process. Additionally, as sulfonamides are amphoteric compounds, their adsorption process is greatly influenced by pH [57]. Therefore, as the pH deviates from its pKa, the sulfonamide’s current form will alter and have an impact on the effectiveness of adsorption. The pka of the six sulfonamides are shown in Table S1. When pH < pKa, sulfonamides mainly exist in the form of cations while when pH > pKa, sulfonamides exist in the form of anions. To study the effect of pH on sulfonamides adsorption, experiments were conducted at pH values ranging from 2 to 10. As shown in Figure 6a, the adsorption capacity of BiFeO_3_/MXene increased with the increase in pH and reached the maximum when the pH was 6 for SDZ, STZ, SMZ, and SMTZ and 5 for SMXZ and SXZ. To further understand the effect of pH on the adsorption of the six sulfonamides, the zeta potential of BiFeO_3_/MXene was investigated, as shown in Figure 6b. It can be seen that BiFeO_3_/MXene reached the isoelectric point (PZC) at a pH of about 2.2. Therefore, BiFeO_3_/MXene was positively charged when the pH was less than 2.2 due to the protonation of surface functional groups, and became negatively charged when the pH was greater than 2.2 due to deprotonation. Due to the existence of oxygen-containing functional groups, with the increase in the pH value, the surface of BiFeO_3_/MXene became deprotonated, and the negative charge on its surface enhanced the electrostatic attraction between the material and positively charged sulfonamides, thereby increasing the adsorption capacity.

#### 2.2.4. Reusability Study

Reusability is one of the most crucial factors in the development of an advanced and efficient adsorbent. Ideally, a promising adsorbent should have a high adsorption capacity and high desorption efficiency, which will lower its overall cost. In this study, after adsorption, the BiFeO_3_/MXene powder was removed from the solution by centrifugation. Then, 1 M NaOH solution was used to desorb the sulfonamides from BiFeO_3_/MXene. To investigate the stability of BiFeO_3_/MXene, SEM, XRD, XPS and FT-IR, characterizations after desorption were performed. The results indicated that there are no diffraction peaks change. In addition, XPS and FT-IR spectra remain unchanged, highlighting the stability of BiFeO_3_/MXene (Figure S4). The recyclability of BiFeO_3_/MXene up to the fifth cycle is shown in Figure S5; it is observed that the adsorption capacity of BiFeO_3_/MXene after 5 regenerations at pH 6 was 7.01, 2.07, 7.18, 7.41, 14.07 and 2.06 mg g^−1^ for SDZ, STZ, SMZ, SMTZ, SMXZ, and SXZ, respectively, and regeneration efficiency of 66.3, 39.2, 70.1, 67.9, 76.8 and 39.6% were achieved for SDZ, STZ, SMZ, SMTZ, SMXZ, and SXZ, respectively, indicating that this adsorbent possesses the capacity of regeneration. However, the decrease in adsorption capacity was due to a reduction in the BiFeO_3_/MXene during the recovery. Therefore, it can be assumed that BiFeO_3_/MXene can be reused for the adsorption of sulfonamides several times with good performance. Moreover, the performance of BiFeO_3_/MXene even after recycling was greater than that of pristine MXene.

#### 2.2.5. Selectivity

BiFeO_3_/MXene was used to remove sulfonamides from actual water samples to test the feasibility of the proposed method. The removal rate of sulfonamides in ultrapure water, tap water, and Yellow-River water by BiFeO_3_/MXene are shown in Figure S6. The results show that removal rates of sulfonamides were in ultrapure water 61.6, 43.9, 72.8, 59.43, 82.5, and 43.09%; in tap water 60.3, 43.2, 71.2, 58.39, 80 and 42.47%; and in Yellow-River water 57.4, 42.04, 69.08, 56.84, 76.7 and 40.5% for SDZ, STZ, SMZ, SMTZ, SMXZ, and SXZ, respectively, were obtained at the concentration of 10 mg L^−1^. This implies a good removal effect on sulfonamides in all samples. In addition, the removal rate follows the order of ultrapure water > tap water > Yellow River water. This may be due to the high level of pollutants competing with sulfonamides for the active sites on the adsorbent in the river water, thus causing a decrease in the sulfonamide removal rate.

## 3. Materials and Methods

### 3.1. Reagents and Materials

Sulfadiazine, 99% (SDZ), sulfathiazole, 99% (STZ), sulfamerazine, 99% (SMZ), sulfamethazine, 99% (SMTZ), sulfamethoxazole, 99% (SMXZ), sulfisoxazole, 99% (SXZ) were supplied by Aladdin Chemical Reagent Company (Shanghai, China). The physicochemical properties and structures of these sulfonamides are shown in Table S1. MAX phase (Ti_3_AlC_2_, 98%) was supplied by Shanghai Macklin Biochemical Co., Ltd. (Shanghai China). Bi(NO_3_)_3_·5H_2_O was supplied by Tianjin Fengyue Chemicals Co., Ltd. (Tianjin, China). Potassium hydroxide (KOH) and sodium hydroxide (NaOH) were supplied by Lian Long Bohua Pharmaceutical Chemical Co., Ltd. (Tianjin, China). Hydrochloric acid (HCl, 32%) was supplied by Sichuan Western science Co., Ltd. (Sichuan, China). Hydrofluoric acid (HF, 40%) and Fe(NO_3_)_3_·9H_2_O were supplied by Chengdu Kelong Chemicals Co., Ltd. (Chengdu, China).

### 3.2. Preparation of Ti_3_C_2_T_x_ MXene

A total of 5.0 g of Ti_3_AlC_2_ powder was dissolved in an 80 mL aqueous solution of hydrofluoric acid (HF, 4%) under vigorous stirring for 24 h at room temperature. This was performed to selectively etch aluminium. Afterward, the obtained product was centrifuged for 10 min at 3500 rpm, and the supernatant was washed with distilled water many times, each time at 3500 rpm for 5 min, to remove the residual HF and impurities until the pH was close to 6. The obtained precipitate (dark green) was dried in an oven at 70 °C for 12 h to obtain the Ti_3_C_2_T_x_ MXene powder.

### 3.3. Preparation of BiFeO_3_/MXene

The BiFeO_3_/MXene was synthesized by a single-step hydrothermal process as shown in Scheme 1. First, 0.01 mol Bi(NO_3_)_3_·5H_2_O and Fe(NO_3_)_3_·9H_2_O were dissolved in 40 mL KOH (10 M) as a mineralizer with a stirring process for 30 min. A total of 0.6 g of MXene were added and continually stirred for 1h. Then, the solution was transferred to a sealed Teflon-lined autoclave and heated at 200 °C for 6 h. The reacted sample was washed 5 times with ethanol and distilled water sequentially, and dried in an oven at 70 °C for 8 h to obtain BiFeO_3_/MXene powder.

### 3.4. Characterizations

The crystal structure was examined using X-ray diffraction diffractometer (X’pert PRO, PANalytical, Almelo, The Netherlands). The BET-specific surface area, pore diameter, and pore size were determined via the N_2_ adsorption–desorption isotherms at 77 K with a surface area (ASAP 2010, Micromeritics, Norcross, Georgia, USA). The morphologies were studied by field emission scanning electron microscope (FESEM) (JSM-6701F scanning electron microscope, JEOL, Tokio, Japan) and transmission electron microscope (TEM) (Tecnai G2TF20, FEI, Hillsboro, Oregon, USA). The functional groups were characterized using Fourier transmission infrared spectroscopy (FTIR) (model IFS120HR, Bruker, Karlsruhe, Germany) equipped with a DTGS detector, collecting 32 scans per sample at a resolution of 4 cm^−1^ and Raman spectroscopy (model IFS120HR, Bruker, Germany). Zeta potential was determined using a Zeta sizer Nano-ZS90 dynamic light scattering instrument (Malvern, Britain). X-ray photoelectron spectroscopy (XPS, ESCALAB 250Xi, Thermo Fisher Scientific, Waltham, Massachusetts, USA) was used to identify the electronic state and chemical bonding at the surface of the synthesized samples. The concentration of sulfonamides was measured by high-performance liquid chromatography (HPLC, 1260 Infinity series, Agilent Technologies, Santa Clara, California, USA).

### 3.5. Adsorption Experiments

The adsorption of sulfonamides on BiFeO_3_/MXene was carried out using a batch experimental process. As a typical adsorption experiment, 20 mg of BiFeO_3_/MXene was suspended in a centrifuge tube containing 10 mL of sulfonamides at pH 6 and was continuously shaken at room temperature for 24 h using a rotary shaker. The samples were then filtered through a 0.22 µm filter and HPLC analysis was performed. The obtained chromatogram is shown in Figure S1. For the isotherm study, the initial concentration of sulfonamides varied from 0 to 100 mg L^−1^ while for the kinetic study, the contact time varied from 0 to 240 min. The adsorbed amounts of sulfonamides were calculated as follows (Equation (6))

(6) $$q_{e}=\frac{\left(c_{o}-c_{e}\right)}{c_{o}}\times V$$

where *q_e_* (mg g^−1^) is the adsorption capacity at equilibrium time, *C_0_* (mg L^−1^) is the concentration of sulfonamides in solution before adsorption, *C_e_* (mg L^−1^) is the concentration of sulfonamides in solution after adsorption, *V* (L) is the volume of sulfonamides solution. 

## 4. Conclusions

A BiFeO_3_/MXene composite was synthesized using a single-step hydrothermal method and was used for the adsorption of sulfonamides from an aqueous solution. The batch adsorption experiments were carried out to study the adsorption of BiFeO_3_/MXene towards sulfonamides and the data were systematically evaluated using kinetic models and isotherm models. The pseudo-second-order model fits the experimental data well, according to kinetic models. In addition, BiFeO_3_/MXene showed the rapid adsorption of sulfonamides and attained equilibrium within 10 min, while the Langmuir model was well-fitted using isotherm models. BiFeO_3_/MXene exhibited an adsorption capacity of 32.7, 54.4, 41.3, 37.8, 29.9, and 24.8 mg g^−1^ for SDZ, STZ, SMZ, SMTZ, SMXZ, and SXZ, respectively, which is higher compared to pristine MXene, BiFeO_3_, and previously reported adsorbent materials. This may be due to the synergistic effect of BiFeO_3_/MXene and MXene. Furthermore, the adsorption behavior was explained mainly by electrostatic attraction between the surface functional groups of BiFeO_3_/MXene and the sulfonamides molecules. The BiFeO_3_/MXene composite had excellent reusability and remarkable selectivity in actual water samples. This research should shed new light on how to design adsorbents that effectively remove sulfonamides from environmental water.

## Figures and Tables

**Table 1:** Fitted parameters of pseudo-first-order and pseudo-second-order models for the adsorption of sulfonamides in a mixed system.

Analyte	*q_e_* _(cal)_ (mg g^−1^)	Pseudo-First-Order Model			Pseudo-Second-Order Model		
		*k*_1_ (min^−1^)	*q_e (exp)_* (mg g^−1^)	*R^2^*	*k*_2_ (g mg^−1^ min^−1^)	*q_e (exp)_* (mg g^−1^)	*R^2^*
SDZ	11.56	−0.0065	9.5	0.966	0.116	10.29	0.999
STZ	5.6	−0.006	5.07	0.939	0.105	3.82	0.999
SMZ	10.94	−0.0073	15.2	0.91	0.074	10.01	0.999
SMTZ	13.15	−0.0074	17.41	0.865	0.077	11.01	0.999
SMXZ	20.12	−0.199	12.59	0.94	0.167	18.99	0.999
SXZ	5.23	−0.0052	3.95	0.864	0.084	4.84	0.999

**Table 2:** Fitted parameters of Langmuir model and Freundlich model for the adsorption of sulfonamides.

			Langmuir Model			Freundlich Model		
Adsorbents	System	Analyte	*q_max_* (mg g^−1^)	*K_L_* (L mg^−1^)	*R^2^*	*n*	*K_F_* (mg g^−1^)/(mg L^−1^)*^n^*	*R^2^*
BiFeO_3_/MXene	single system	SDZ	32.68	6.027	0.983	1.79	4.924	0.941
		STZ	54.45	1.651	0.999	1.24	1.856	0.989
		SMZ	41.27	1.247	0.997	1.30	1.476	0.981
		SMTZ	37.80	1.106	0.999	1.34	1.387	0.987
		SMXZ	29.87	6.903	0.997	2.03	5.715	0.925
		SXZ	24.81	10.00	0.999	2.43	6.440	0.875
	mixed system	SDZ	11.56	1.063	0.994	1.9	1.264	0.963
		STZ	5.60	0.632	0.997	2.13	0.784	0.908
		SMZ	10.94	1.407	0.991	2	1.478	0.840
		SMTZ	13.15	0.816	0.993	1.66	0.990	0.969
		SMXZ	20.12	4.063	0.990	2.28	3.530	0.968
		SXZ	5.23	0.736	0.999	2.40	1.090	0.938
MXene	mixed system	SDZ	4.94	0.948	0.995	3.33	1.318	0.960
		STZ	3.05	0.298	0.998	2.23	0.439	0.891
		SMZ	4.28	0.254	0.996	1.83	0.373	0.958
		SMTZ	6.90	0.542	0.995	1.98	0.756	0.970
		SMXZ	7.07	0.676	0.996	1.98	0.838	0.950
		SXZ	1.46	0.599	0.999	4.55	0.603	0.780
BiFeO_3_	mixed system	SDZ	4.89	0.822	0.996	2.94	1.122	0.968
		STZ	2.84	0.230	0.994	2.05	0.339	0.867
		SMZ	3.85	0.165	0.987	1.62	0.234	0.950
		SMTZ	5.23	0.399	0.982	2.15	0.643	0.965
		SMXZ	5.73	0.481	0.990	2.12	0.710	0.975
		SXZ	1.30	0.583	0.997	4.64	0.549	0.843

**Table 3:** The comparison of maximum adsorption capacities of various adsorbents for sulfonamides adsorption. Benzene-1,3,5-tricarbohydrazide reacted with benzidine (JLUE-COP-26), magnetic-reduced graphene oxide (MrGO), enzyme-induced carbonate precipitation (EICP), Triformylphloroglucinol p-Phenylenediamine (TpPa-1), 1,3,5-triformylbenzene-Benzidine (TFBBD), zeolitic imidazolate framework-8 (ZIF-8), and primary paper-mill sludge (PS800-150).

Sulfonamide	Adsorbent	System	*q_max_* mg g^−1^	References
SDZ	Hydrochar	Single system	0.12	[51]
	Expanded graphite	Single system	16.58	[52]
	JLUE-COP-26	Single system	10.01	[53]
	MrGO	Single system	6.27	[54]
	BiFeO_3_/MXene	Single system	32.68	This work
	BiFeO_3_/MXene	Mixed with STZ, SMZ, SMTZ, SMZX, and SXZ	11.56	This work
STZ	Sewage sludge	Mixed with sulfadiazine	8.52	[55]
	EICP	Single system	4.92	[8]
	Functionalized biochar	Mixed with sulfamethazine, sulfamethoxazole, and chloramphenicol	45.19	[56]
	BiFeO_3_/MXene	Single system	54.45	This work
	BiFeO_3_/MXene	Mixed with SDZ, SMZ, SMTZ, SMZX, and SXZ	5.60	This work
SMZ	TpPa-1	Mixed with sulfamethazine, sulfamonomethoxine, sulfamethoxazole, and sulfadimethoxine	2.2	[57]
	Fe/Ni-PG	Single system	27.3	[58]
	BiFeO_3_/MXene	Single system	41.27	This work
	BiFeO_3_/MXene	Mixed with SDZ, STZ, SMTZ, SMZX, and SXZ	10.94	This work
SMTZ	Modified activated carbon	Single system	17.24	[59]
	TFBBD	Mixed with Sulfamerazine, sulfamonomethoxine, sulfamethoxazole, and sulfadimethoxine	10.1	[57]
	BiFeO_3_/MXene	Single system	37.8	This work
	BiFeO_3_/MXene	Mixed with SDZ, STZ, SMZ, SMZX, and SXZ	13.15	This work
SMXZ	ZIF-8	Single system	21.9	[60]
	Phosphorus-containing stalk cellulose	Mixed with SDZ	2.52	[61]
	Pristine capped CNTs	Mixed with sulfadimethoxine, sulfamethizole, and sulfamethazine	7.88	[62]
	PS800-150	Single system	1.69	[63]
	BiFeO_3_/MXene	Single system	29.87	This work
	BiFeO_3_/MXene	Mixed with SDZ, STZ, SMZ, SMTZ, and SXZ	20.12	This work
SXZ	Fe_2_O_3_@SiO_2_	Mixed with sulfisoxazole, sulfadimethoxine, trimethoprim, and sulfamerazine	2.76	[64]
	BiFeO_3_/MXene	Single system	24.81	This work
	BiFeO_3_/MXene	Mixed with SDZ, STZ, SMZ, SMTZ, and SMZX	5.23	This work

## Data Availability

Not applicable.

## Supplementary Materials

The following supporting information can be downloaded at: https://www.mdpi.com/article/10.3390/molecules28041541/s1, Figure S1:Chromatogram of the six sulfonamides; Figure S2: N_2_ adsorption-desorption isotherms of (a) MXene and (b) BiFeO_3_/MXene, insert is their corresponding pore size distribution; Figure S3: (a) Linear Langmuir and (b) Freundlich fitting curves for sulfonamides adsorption on BiFeO_3_/MXene in a single system; Figure S4: SEM of BiFeO_3_/MXene after desorption; Figure S5: The recyclability of BiFeO_3_/MXene on adsorption of sulfonamides; Figure S6: Removal rate of BiFeO_3_/MXene on sulfonamides; Table S1: Selected characteristics of the six sulfonamides.
