# Supplementary material for: Effective Removal of Sulfonamides Using Recyclable MXene-Decorated Bismuth Ferrite Nanocomposites Prepared via Hydrothermal Method
Source: Molecules. 2023 Feb 5;28(4):1541. doi: 10.3390/molecules28041541 (PMC9962683; doi:10.3390/molecules28041541)
Supplement: Supplementary file 1 [file molecules-28-01541-s001.zip › molecules-2144675-supplementary.pdf]

Effective removal of sulfonamides using recyclable MXene decorated bismuth ferrite nanocomposites prepared via hydrothermal method

Pascaline Sanga [a,b], Juanjuan Wang [a], Xin Li [a], Jia Chen [a], Hongdeng Qiu [a,b,c,*]

[a] CAS Key Laboratory of Chemistry of Northwestern Plant Resources and Key Laboratory for Natural Medicine of Gansu Province, Lanzhou Institute of Chemical Physics, Chinese Academy of Sciences, Lanzhou 730000, China.

[b] University of Chinese Academy of Sciences, Chinese Academy of Sciences, Beijing 100039, China.

[c] College of Chemistry, Zhengzhou University, Zhengzhou 450001, China.

[*] Corresponding e-mail: hdqiu@licp.cas.cn (H. Qiu)

HPLC Conditions

The concentrations of the six sulfonamides were analyzed by HPLC (1260 Infinity series, Agilent Technologies, USA) using a ZORBAX SB-C18 column (5 μm, 4.6 mm×250 mm i.d.) as a stationary phase and UV detector. The mobile phase was D-0.2% acetic acid aqueous solution, C-methanol, The gradient elution procedure was 0-3 min:10% methanol; 3-4 min:10%-20% methanol; 4 min-8 min 20 % methanol; 8 min-15 min 20%-30% methanol; 15 min-17 min 30%~45% methanol and the wavelength of the UV detector was 250 nm. The flow rate was 1 mL/ min, and the column temperature was 25 °C. The injection volume was 10 μL.

Table S1. Selected characteristics of the six sulfonamides

Name	Molecular structure	Molecular formula	Molecular weight (g/mol)	pKa	Water Solubility (mg/mL)
Sulfadiazine	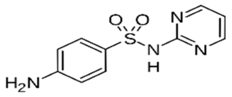	$C_{10}H_{10}N_4O_2S$	250.277	6.41±0.14	4.12
Sulfathiazole	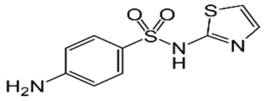	$C_9H_9N_3O_2S_2$	255.317	7.15±0.12	0.921
Sulfamerazine	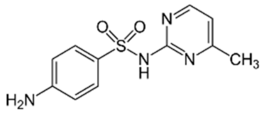	$C_{11}H_{12}N_4O_2S$	264.304	6.92±0.14	0.304
Sulfamethazine	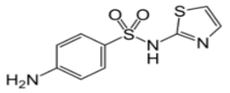	$C_{12}H_{14}N_4O_2S$	278.33	7.56±0.26	0.23
Sulfamethoxazole	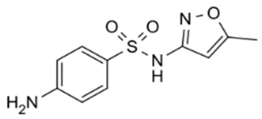	$C_{10}H_{11}N_3O_3S$	253.278	5.73±0.20	0.459
Sulfisoxazole	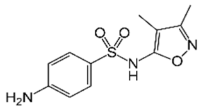	$C_{11}H_{13}N_3O_3S$	267.304	5.00±0.00	0.313

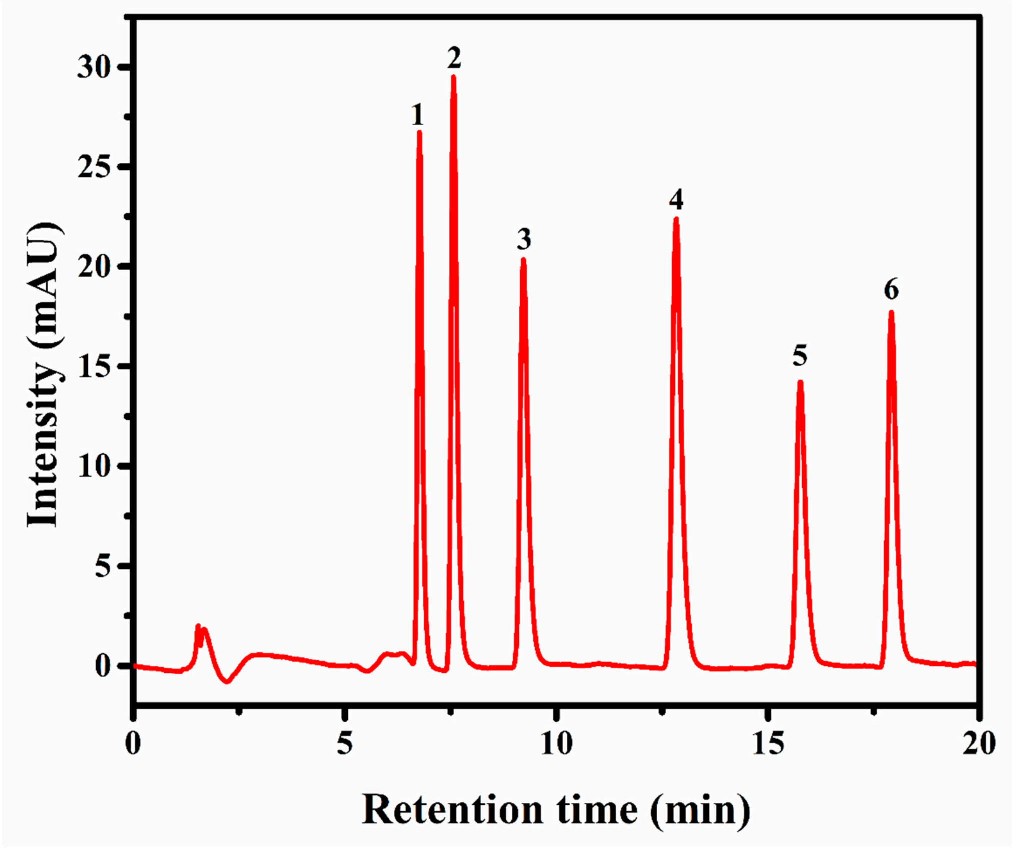

Figure S1. Chromatogram of the six sulfonamides. sulfadiazine (1), sulfathiazole (2), sulfamerazine (3), sulfamethazine (4), sulfamethoxazole (5), and sulfisoxazole (6).

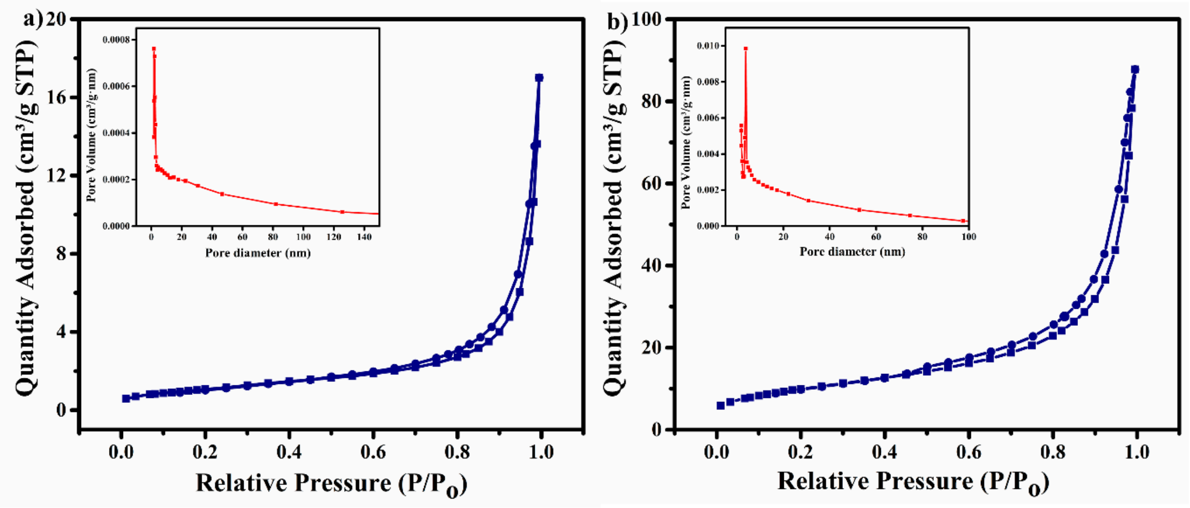

Figure S2. N_2 adsorption-desorption isotherms of a)MXene and b)$BiFeO_3$/MXene, insert is their corresponding pore size distribution.

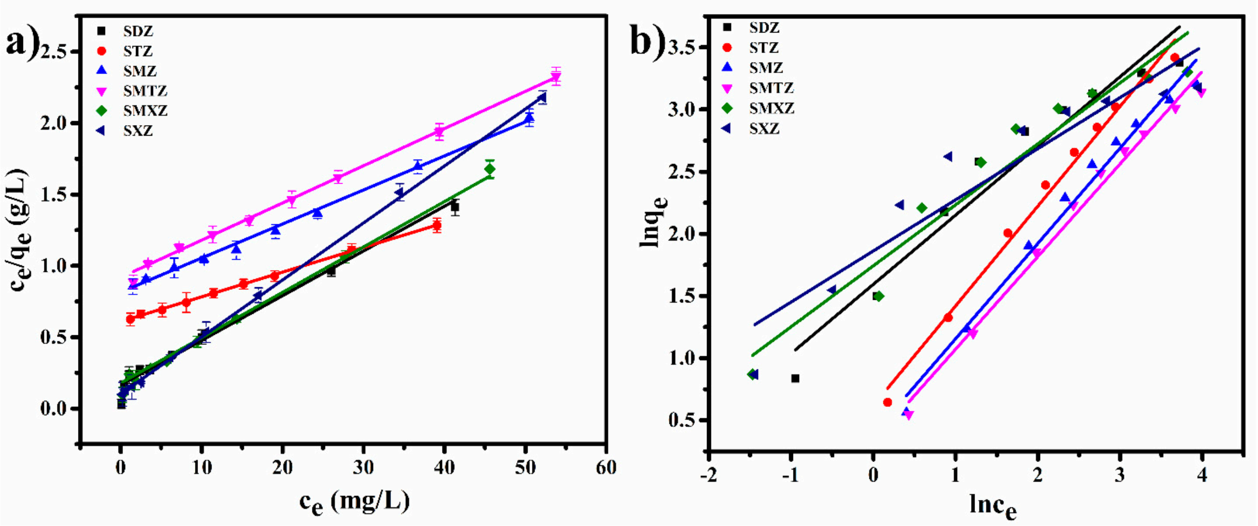

Figure S3. a) Linear Langmuir and b) Freundlich fitting curves for sulfonamides adsorption on $BiFeO_3$/MXene in a single system at pH of 6 for SDZ, STZ, SMZ, and SMTZ, and pH of 5 for SMXZ and SXZ.

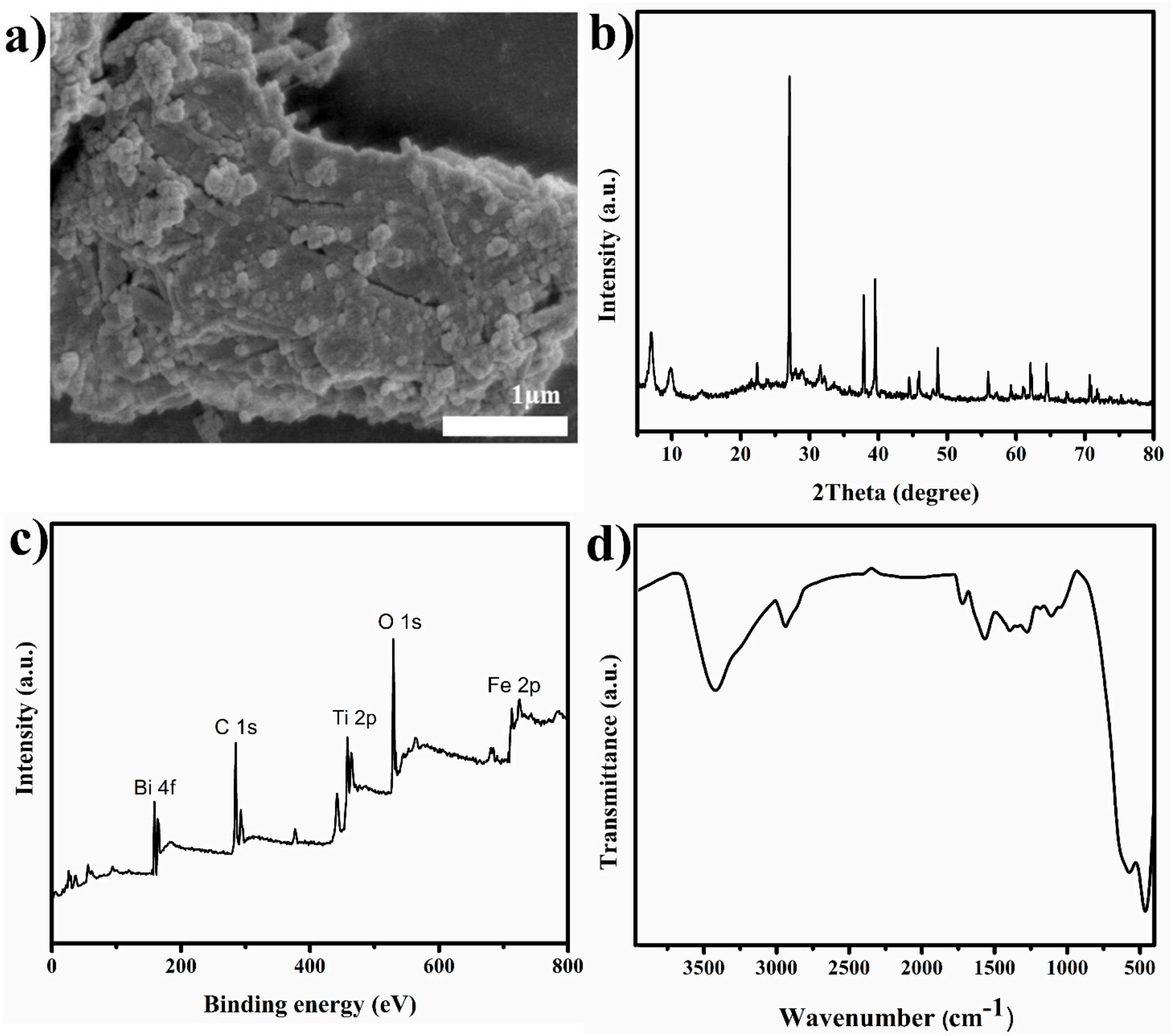

Figure S4. a) SEM, b) XRD, c)XPS, and d) FT-IR of $BiFeO_3$/MXene after desorption.

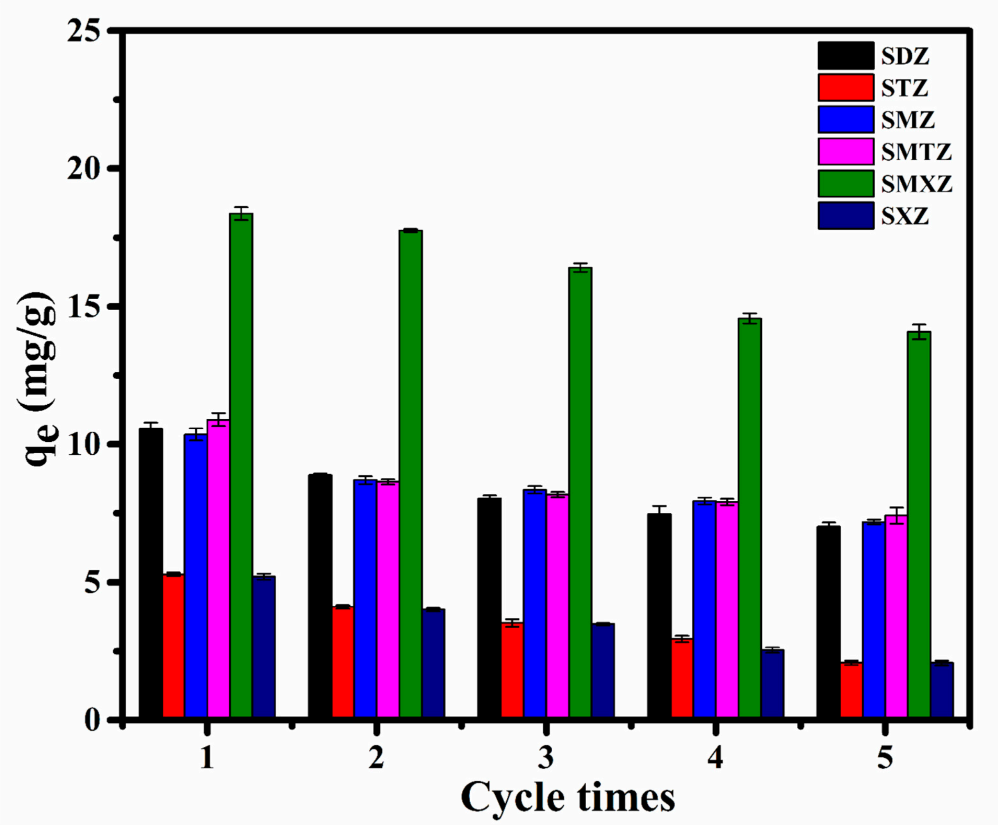

Figure S5. The recyclability of $BiFeO_3$/MXene on adsorption of sulfonamides.

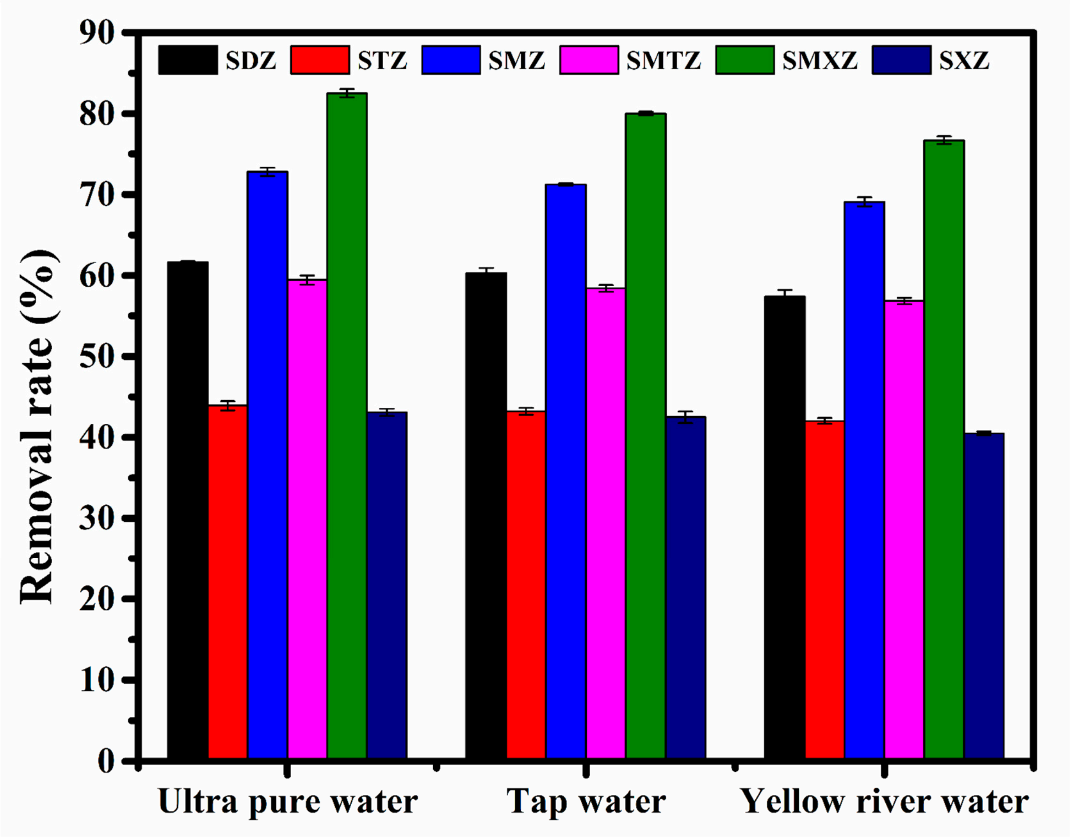

Figure S6. Removal rate of $BiFeO_3$/MXene on sulfonamides.
